# Urinary Metabolomic Markers of Protein Glycation, Oxidation, and Nitration in Early-Stage Decline in Metabolic, Vascular, and Renal Health

**DOI:** 10.1155/2019/4851323

**Published:** 2019-11-19

**Authors:** Jinit Masania, Gernot Faustmann, Attia Anwar, Hildegard Hafner-Giessauf, Nasir Rajpoot, Johanna Grabher, Kashif Rajpoot, Beate Tiran, Barbara Obermayer-Pietsch, Brigitte M. Winklhofer-Roob, Johannes M. Roob, Naila Rabbani, Paul J. Thornalley

**Affiliations:** ^1^Warwick Medical School, Clinical Sciences Research Laboratories, University of Warwick, University Hospital, Coventry CV2 2DX, UK; ^2^Clinical Division of Nephrology, Department of Internal Medicine, Medical University of Graz, 8036 Graz, Austria; ^3^Human Nutrition & Metabolism Research and Training Center (HNMRC), Institute of Molecular Biosciences, Karl Franzens University of Graz, Universitätsplatz 2, 8010 Graz, Austria; ^4^Department of Computer Sciences, University of Warwick, Coventry CV4 7AL, UK; ^5^School of Computer Science, University of Birmingham, Edgbaston, Birmingham B15 2TT, UK; ^6^Clinical Institute of Medical and Clinical Laboratory Diagnostics, Medical University of Graz, 8036 Graz, Austria; ^7^Clinical Division of Endocrinology, Department of Internal Medicine, Medical University of Graz, 8036 Graz, Austria; ^8^Diabetes Research Center, Qatar Biomedical Research Institute (QBRI), Hamad Bin Khalifa University, Qatar Foundation, P.O. Box 34110, Doha, Qatar

## Abstract

Glycation, oxidation, nitration, and crosslinking of proteins are implicated in the pathogenic mechanisms of type 2 diabetes, cardiovascular disease, and chronic kidney disease. Related modified amino acids formed by proteolysis are excreted in urine. We quantified urinary levels of these metabolites and branched-chain amino acids (BCAAs) in healthy subjects and assessed changes in early-stage decline in metabolic, vascular, and renal health and explored their diagnostic utility for a noninvasive health screen. We recruited 200 human subjects with early-stage health decline and healthy controls. Urinary amino acid metabolites were determined by stable isotopic dilution analysis liquid chromatography-tandem mass spectrometry. Machine learning was applied to optimise and validate algorithms to discriminate between study groups for potential diagnostic utility. Urinary analyte changes were as follows: impaired metabolic health—increased N_*ε*_-carboxymethyl-lysine, glucosepane, glutamic semialdehyde, and pyrraline; impaired vascular health—increased glucosepane; and impaired renal health—increased BCAAs and decreased N_*ε*_-(*γ*-glutamyl)lysine. Algorithms combining subject age, BMI, and BCAAs discriminated between healthy controls and impaired metabolic, vascular, and renal health study groups with accuracy of 84%, 72%, and 90%, respectively. In 2-step analysis, algorithms combining subject age, BMI, and urinary N_*ε*_-fructosyl-lysine and valine discriminated between healthy controls and impaired health (any type), accuracy of 78%, and then between types of health impairment with accuracy of 69%-78% (*cf.* random selection 33%). From likelihood ratios, this provided small, moderate, and conclusive evidence of early-stage cardiovascular, metabolic, and renal disease with diagnostic odds ratios of 6 – 7, 26 – 28, and 34 – 79, respectively. We conclude that measurement of urinary glycated, oxidized, crosslinked, and branched-chain amino acids provides the basis for a noninvasive health screen for early-stage health decline in metabolic, vascular, and renal health.

## 1. Introduction

Diabetes mellitus, cardiovascular disease (CVD), and chronic kidney disease (CKD) are major noncommunicable chronic diseases in adults linked to premature death and loss of productive life in Westernised countries. Type 2 diabetes mellitus (T2DM) linked to development of insulin resistance and dysglycemia in prediabetes accounts for *ca.* 90% of cases of diabetes [1]. Spontaneous and potentially damaging modifications of proteins by reactive oxygen species (ROS) have been implicated in the pathogenesis of this disease development [2–4]. Protein glycation by glucose to form fructosamine adducts, particularly as assessed by glycated hemoglobin A1C, is a major clinical measure of glycemic control in diabetes and at lower levels is considered a diagnostic marker of prediabetes [5]. More recently, advanced glycation end products (AGEs), formed by the degradation of proteins glycated by glucose and by the direct reaction of proteins with reactive dicarbonyl compounds such as methylglyoxal, have been proposed as both mediators of health decline leading to T2DM, CVD, and CKD [6–8]. Examples of the latter are association of fructosamine-derived AGE, glucosepane, with development of T2DM [6] and association of increased formation of the methylglyoxal-derived AGE, hydroimidazolone MG-H1, with insulin resistance, risk of CVD, and development of CKD [9–11]. Oxidized and glycated proteins are targeted for cellular proteolysis which forms related oxidized and glycated amino acid metabolites, also called protein oxidation and glycation free adducts. These are released from cells and excreted in urine [12]. Oxidized and glycated amino acids are also absorbed from the intestinal tract after digestion of oxidized and glycated proteins of ingested food [13]. Pyrraline, an AGE formed only at the high temperatures of culinary processing of food, is absorbed and is a marker of the dietary AGEs [9]. Measurement of urinary fluxes of oxidized and glycated amino acids gives an estimate of total body exposure to these adducts—except for N_*ε*_-fructosyl-lysine (FL) which may be further metabolised enzymatically [14].

Irreversible protein crosslinking increases with age and may contribute mechanistically to the related age-associated increased risk of T2DM, CVD, and CKD [1, 15]. Dityrosine is a major oxidative crosslink, particularly through enzymatic formation by dual oxidase (DUOX) [16], glucosepane is a major glycation-derived protein crosslink [17], and N_*ε*_-(*γ*-glutamyl)lysine (GEEK) is a crosslink formed in proteins catalysed by transglutaminases [18]. Dityrosine and GEEK may also be absorbed from digested proteins in food [19, 20]. Trace level urinary fluxes of oxidized, glycated, and crosslinked amino acids reflect the flux of formation of glycated, oxidized, and crosslinked proteins—with also contributions from food. We hypothesized that early-stage changes in urinary fluxes of oxidized, glycated, crosslinked, and branched-chain amino acids may provide biomarkers supporting the early-stage diagnosis of impaired metabolic, vascular, and renal disease. Branched-chain amino acids (BCAAs) have been linked previously to the development of T2DM and CKD [21, 22].

In this study, we determined urinary fluxes of oxidized, glycated, crosslinked, and branched-chain amino acids in healthy human subjects and subjects with early-stage decline in metabolic, vascular, and renal health, exploring potential diagnostic utility by data-driven machine-learning approaches. We based our sample collection and analysis on urine as a sample type favoured for clinical metabolomic applications because of the advantages of it being a readily available sample, ease of sample donation and collection, and less complex sample matrix than other body fluids such as serum or plasma. Oxidized, glycated, and crosslinked amino acids determined were as follows: FL—the major early-stage protein glycation adduct formed by glucose; MG-H1 and N_*ε*_-carboxymethyl-lysine (CML)—the major AGEs; pyrraline—a marker of dietary AGE exposure and absorption [23, 24]; protein crosslinks—glucosepane, pentosidine, dityrosine, and GEEK; glutamic acid semialdehyde (GSA)—a “protein carbonyl” marker of oxidative damage; and 3-nitrotyrosine—a marker of protein nitration [25]. Protein modification analysis classes and associated processes reported thereby are summarised in Table 1.

## 2. Materials and Methods

### 2.1. Subject Study Groups and Sampling

A total of 200 study participants of the BIOCLAIMS cohort, recruited and investigated at the Medical University and Karl Franzens University of Graz, Austria, between May 2011 and November 2014, were investigated in this study. This cohort was conceived, subjects were recruited, and samples were collected as part of the EU FP7 BIOCLAIMS research project. The underlying hypothesis of this project was that maintenance of good health may be improved with biomarkers of good health or “health biomarkers” [26, 27]. We hypothesized that the range of flux of protein damage by glycation, oxidation, nitration, and crosslinking found in subjects of good health, singly or combinations thereof, may serve as health biomarkers and this could be tested by studying the changes in fluxes of related analytes in early decline in health—representing a challenge to health homeostasis. We selected early decline in metabolic, vascular, and renal health as examples of major health impact through further progression to type 2 diabetes, CVD, and renal failure. Measurement of urinary glycated, oxidized, nitrated, and crosslinked amino acids provide a surrogate measure of this. Changes in these analytes may thereby be used to diagnose early-stage decline of health. The subjects were assigned to one of the four groups.

#### 2.1.1. Group 1: Healthy Controls (*n* = 55)

The inclusion criteria for these subjects were as follows: BMI 18.5-29.9 kg/m^2^, carotid artery intimal medial thickness (CIMT) ≤ 75^th^ percentile at the left plus right side, homeostatic model assessment of insulin resistance (HOMA-IR) index ≤ 2.5 mUl^−1^ mM, A1C < 38 mmol/mol, (or either HOMA-IR or A1C was allowed above this threshold but not both) estimated glomerular filtrate rate (eGFR) > 60 ml/min/1.73 m^2^ (deduced from increased serum creatinine by the Modification of Diet in Renal Disease Study equation [28]), and clinical chemistry tests within the normal range ± 10%. The clinical chemistry tests included the following: red and white blood cell counts, hemoglobin, hematocrit, thrombocyte count, serum electrolyte, creatinine, urea, uric acid and cystatin C concentrations, *γ*-glutamyl-transpeptidase, cholinesterase, aspartate aminotransferase, alanine aminotransferase activity, pancreatic amylase and lipase activity, serum fasting glucose, C-reactive protein, total cholesterol, HDL and LDL cholesterol, triglyceride, apolipoprotein-A1 and apolipoprotein-B, total protein, albumin, iron, transferrin and ferritin concentrations, and thyroid gland stimulating hormone activity.

#### 2.1.2. Group 2: Mild Impairment of Metabolic Health (*n* = 44)

The inclusion criteria for these subjects were as follows: HOMA-IR index > 2.5 [29] and A1C 38-46 mmol/mol [30] with eGFR > 60 ml/min/1.73 m^2^ and CIMT ≤ 75^th^ percentile at the left plus right side.

#### 2.1.3. Group 3: Mild Impairment of Vascular Health (*n* = 58)

The inclusion criteria for these subjects were as follows: CIMT > 75^th^ percentile for age and sex on the left and right sides [31] with eGFR > 60 ml/min/1.73 m^2^, HOMA-IR index ≤ 2.5, and A1C < 38 mmol/mol (or either HOMA-IR or A1C was allowed above this threshold but not both).

#### 2.1.4. Group 4: Mild Impairment of Renal Health (*n* = 43)

The inclusion criteria for these subjects were as follows: an eGFR of 30-60 ml/min/1.73 m^2^ and serum cystatin C > 1.04 mg/l (upper limit of normal range) with HOMA-IR index ≤ 2.5 mUl^−1^ mM and A1C < 38 mmol/mol (or either HOMA-IR or A1C was allowed above this threshold but not both) and CIMT ≤ 75^th^ percentile at the left plus right side [32, 33]. Their etiology of kidney disease was other than diabetic kidney disease which was excluded to meet the inclusion criterion of glycemic control in this study group.

Peripheral venous blood samples were collected on Vacutainer tubes, coated with EDTA, heparin or none after overnight fasting. Heparin plasma and serum were obtained by centrifugation (1620g, 10 min), and red blood cells were separated from EDTA whole blood by centrifugation (10000g, 1 min) and washed with NaCl. Clinical chemistry routine variables were analysed the same day, while the other aliquots were stored at −80°C until analysis. A urine sample, second void after overnight fasting, was collected, centrifuged to sediment cells present, and supernatant removed and stored at −80°C until analysis. Urine samples were collected in the second void after overnight fasting to decrease contributions of glycated, oxidized, and nitrated amino acids from digested proteins in food [34].

The collection of samples from subjects with written informed consent, use of them, and study protocols were approved by the Ethics Committee of the Medical University and Karl Franzens University of Graz, Austria, and were conducted in accordance with the Declaration of Helsinki.

### 2.2. Measurement of Urinary Glycated, Oxidized, Nitrated, Crosslinked, and Branched-Chain Amino Acids

Amino acid analytes were quantified in the ultrafiltrate of second void urine after overnight fast by LC-MS/MS multiple reaction monitoring (MRM) as described previously [12, 35] with detection of additional analytes GEEK, leu, ile, and val. Urinary analytes were normalised to urinary creatinine because spot urine samples were used. Urine samples were filtered through 3 kDa pore size microspin filters (14,000 g, 4°C). Ultrafiltrate (5 *μ*l) was mixed with a cocktail of stable isotopic standards (25 *μ*l) and analysed by LC-MS/MS using an Acquity™ ultrahigh performance liquid chromatography-Xevo-TQS LC-MS/MS system (Waters, Manchester, U.K.). MRM detection conditions are summarised with chromatographic conditions, calibration, limit of detection, analytical recovery, and inter- and intrabatch coefficient of variance as given previously [35] (Table 2). GEEK was detected in a separate chromatography run to resolve it from isobaric glu-lys and lys-glu dipeptides [36]. Analyte amounts in test samples were deduced by interpolation of analyte/internal standard peak area ratios deduced from MRM mass chromatograms on calibration curves constructed by analysis of calibration standards.

Natural isotopic abundance analytical standards and stable isotope-substituted internal standards were purchased (Sigma-Aldrich, Poole, Dorset, U.K., and Cambridge Isotope Laboratories, Tewksbury, MA, USA) or synthesised in-house where unavailable commercially, as previously described [12, 37]. GSA was prepared from N-acetyl-L-ornithine using lysyl oxidase activity from eggshell membrane by modification of the method of Akagawa et al. [38]. Briefly, eggshell membranes (ESM) were isolated from 12 fresh hen eggs, washed thoroughly with distilled water, and cut into small pieces (5 × 5 mm). Surface water was blotted from ESM with a filter paper. N_*α*_-Acetyl-L-ornithine (43.6 mg, 0.25 mmol) was incubated with ESM (2.5 g) in 25 ml sodium phosphate buffer (20 mM, pH 9.0, 37°C) for 7 days with shaking. The eggshell membranes were removed by centrifugation (6000g, 10 min, 20°C), and the reaction mixture adjusted to pH 7.0. Acylase-1 (20 mg; grade I from porcine kidney, ≥2,000 units/mg protein, Sigma-Aldrich, Cat no. A3010) was added, and the reaction mixture was incubated at 25°C for 2.5 days. The reaction mixture was filtered (3 kDa) to remove acylase and the resulting GSA solution used without further purification. GSA was calibrated by derivatisation with 2-aminobenzaldehyde (OBA) to a dihydroquinazolinium adduct [39] for which the extinction coefficient of 2,800 M^−1^ cm^−1^ was assumed (as for related *α*-aminoadipic semialdehyde adduct) [40]. GSA solution was incubated with 15 mM OBA in water at 20°C for 20 min and absorbance measured at 465 nm. The yield of GSA was 83%. The internal standard used for GSA was [^2^H_3_]*α*-aminoadipic acid ([^2^H_3_]AAA; C/D/N Isotopes Inc., Pointe-Claire, Quebec, Canada) [41]. Stable isotopic N_*ε*_-(*γ*-[^13^C_5_]glutamyl)lysine ([^13^C_5_]GEEK) was prepared by modification of the facile synthesis described previously [42]. *L*-[^13^C_5_]Glutamic acid (5.0 mg, 34 *μ*mol) and *L*-lysine (5.0 mg, 34 *μ*mol) were suspended in pentan-1-ol (25 *μ*l), methanol (2 *μ*l), and water (2 *μ*l) in a 0.3 ml reaction vial. The mixture was heated at 130°C for 5 h. The solvent was removed under reduced pressure, and the residues were lyophilised to dryness to give [^13^C_5_]GEEK, 6.8 mg (yield: 71%) which was used without further purification.

### 2.3. Machine Learning

Support vector machine (SVM) learning methods were used to produce classification algorithms [43]. Only urinary analytes, age, BMI, and gender were included as features to limit analysis sample requirement to urine only. These were developed to discriminate between study groups in two different setups. In the first setup, SVMs were used as a supervised machine learning binary classifier that develops a model from the training data to discriminate between the healthy and a diseased class using a linear hyperplane in a high-dimensional space. Subsequently, the trained model is applied to unseen data to predict the study group class of the related subject. In the second setup, we developed a two-step algorithm. In step 1, the algorithm was trained to discriminate between healthy and diseased state where all disease impairments were considered as a disease class. In the second step, the algorithm was trained to identify one of the three disease impairments. For the three-class system explored (discriminating between impaired metabolic, vascular, and renal health study groups), the trained algorithm classifies the subject into one of three groups. For all classification experiments, we used a 2-fold cross-validation system where data is split into 50% each for training and testing and then this data split is alternated. This was repeated 100 times to test the stability and generalizability of the classification system. To assess the diagnostic characteristics, 8 performance metrics were computed: classification accuracy, sensitivity, specificity, positive likelihood rate, negative likelihood rate, positive predictive value, negative predictive value, and F-measure/score. The false positive rate is also described (=1 − specificity). The 95% CI are determined by Student's *t* distribution.

### 2.4. Statistical Analyses

Data are presented as mean ± SD for parametric distributions and median (lower-upper quartile) for nonparametric distributions. For two groups, significance of the difference between means of parametric data was analysed by Student's *t*-test and medians of nonparametric data by the Mann-Whitney *U* test for independent samples; for more than two groups, significance of the difference between means of parametric data was analysed by ANOVA and medians of nonparametric data by the Kruskal-Wallis test for independent samples. Correlation analysis was performed by the Spearman rank correlation method. Data were analysed using SPSS, version 24.0. *P* < 0.05 was considered significant.

## 3. Results

### 3.1. Clinical Characteristics of Subjects Recruited

Characteristics of the subjects recruited with and without early-stage impairment of metabolic, vascular, and renal health are given (Table 3). Healthy control subjects without impaired metabolic, vascular, and renal health were younger and had lower BMI, A1C, and CIMT than study groups with impaired health. All health impairment study groups had increased plasma total cholesterol with respect to healthy controls, with increased LDL cholesterol in impaired metabolic and vascular health and decreased HDL cholesterol and increased triglycerides and systolic and diastolic blood pressure in impaired vascular and renal health. Urinary albumin and total protein were increased only in impaired renal health, with respect to healthy controls. Changes in plasma LDL cholesterol and HDL cholesterol were not always accompanied by similar changes in plasma ApoB and ApoA1, respectively.

### 3.2. Urinary Amino Acid Metabolites

Urinary levels of glycated, oxidized, nitrated, crosslinked, and branched amino acid analytes for the 4 study groups are given in Table 4. Urinary fluxes of modified amino acids in healthy controls were in the order: FL>CML>MG-H1≈pyrraline>GSA>glucosepane>GEEK>DT>3-NT. For protein crosslinks, levels of urinary fluxes were in the order: glucosepane>GEEK>pentosidine>DT. For BCAAs, urinary excretion was in the order: val>leu>ile. With respect to healthy controls, changes found were as follows: impaired metabolic health—increased urinary excretion of CML, glucosepane, pyrraline, and GSA; impaired vascular health—increased glucosepane; and impaired renal health—increased BCAAs and decreased GEEK.

In correlation analysis, for healthy control subjects, there was no association of any urinary biomarkers with subject age. FL correlated positively with MG-H1 (*r* = 0.84) and 3-NT (*r* = 0.62), and MG-H1 correlated positively with 3-NT (*r* = 0.72). BCAAs correlated positively with each other (*r* = 0.74–0.82). There were also positive correlations of pyrraline with FL, CML, and MG-H1 (*r* = 0.43), suggesting significant contributions to urinary fluxes of these analytes from food—[Supplementary-material supplementary-material-1]. In subjects with impaired metabolic health, surprisingly, there were no correlations of A1C or HOMA-IR with urinary glycation adducts. There were negative correlations of all BCAAs with A1C (*r* = −0.38 to −0.39) and positive correlations of BCAAs with eGFR (*r* = 0.38–0.50), GSA (*r* = 0.57–0.61), and GEEK (*r* = 0.45–0.54). There were positive correlations of pyrraline with CML, MG-H1, GSA, and GEEK (*r* = 0.44–66), suggesting these analytes had significant contributions from food—[Supplementary-material supplementary-material-1]. In subjects with impaired vascular health, there were positive correlations of CIMT, A1C, and glucosepane with age (*r* = 0.86, *r* = 0.51, and *r* = 0.37, respectively) and negative correlation of eGFR with age (*r* = −0.51). Subjects with impaired vascular health, therefore, have age-related increase in CIMT thickness along with age-related early-stage decline in glucose tolerance and renal function and increased glucose-mediated protein crosslinking. There were positive correlations of pyrraline with CML (*r* = 0.57), MG-H1 (*r* = 0.42), GSA (*r* = 0.49), and DT (*r* = 0.39), suggesting these analytes had significant contributions from food—[Supplementary-material supplementary-material-1]. In subjects with impaired renal health, there were positive correlations of CIMT (*r* = 0.79) and glucosepane with age (*r* = 0.46), suggesting that subjects with impaired renal function have increased CIMT and increased glucose-mediated protein crosslinking with age. There were positive correlations of pyrraline with CML (*r* = 0.50) and MG-H1 (*r* = 0.55), suggesting again these AGE free adducts had significant contributions from food—[Supplementary-material supplementary-material-1].

### 3.3. Machine Learning Analysis

We performed machine learning-based analysis on subject groups with and without early-stage decline in metabolic, vascular, and renal health. Several methods for subject assignment to one of the above four groups were tested with the SVM method producing superior diagnostic performance [43]. Features included in algorithm training were as follows: age, gender, BMI, alcohol intake, smoking status (current, former smoker, and never smoked), and urinary metabolites—FL, CML, MG-H1, GSP, pentosidine, pyrraline, DT, GSA, 3-NT, GEEK, and leu, ile, and val or total BCAA. Selection of optimal subset of features was data-driven to those combinations that minimise the group assignment error rate.

In the initial computations, we trained algorithms for each health impairment and healthy subjects. Few features were required for optimum diagnosis outcome. We found three combinations of features, Sets 1 – 3, which gave similar diagnostic performance: Set 1 features—age, BMI, and urinary excretion of FL and valine; Set 2 features—age, BMI, and urinary excretion of leu; and Set 3 features—age, BMI, and urinary excretion of val (Table 5). Diagnostic accuracies were 71 – 90%, sensitivities 68 – 88%, and specificities 71 – 92%. The highest accuracies for impaired metabolic, vascular, and renal health were 84%, 72%, and 90%, respectively, with lowest false positive rates of 13%, 24%, and 8%. The highest positive likelihood ratios for impaired metabolic, vascular, and renal health were 8.0, 3.2, and 13.2, respectively, indicating that this biomarker set gave moderate evidence of impaired metabolic health, limited evidence of impaired vascular health, and convincing evidence of impaired renal health [44].

**Figure 1:**
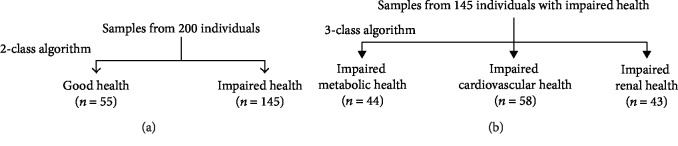
Training and validation of a multiclass algorithm for detection and discrimination of (a) good health versus impaired health disease and (b) impaired metabolic, vascular, and renal health.

As a second approach, we performed a two-step algorithm analysis where step 1 distinguished between good health and impaired health of any kind investigated and then step 2 distinguished between the three different types of impaired health—metabolic, vascular, or renal (Figure 1). In step 1, outcome of the algorithm computations indicated that again few features were required for this: age, BMI, and urinary excretion of FL and valine. For step 1, the accuracy was 78% (random selection = 0.50) and sensitivity/specificity was 82/77% (Table 6). The positive likelihood ratio was 3.7, indicating the test provides moderate evidence of health impairment. In step 2, a 3-class algorithm was produced to distinguish between metabolic, vascular, or renal health impairment. Outcome of the algorithm computations indicated that features required for this are as follows: age, BMI, and urinary excretion of val. The accuracy for step 2 was 69–78% (random selection = 0.33). The positive likelihood ratios were in the range of 2.6 – 4.6, indicating a small increase in likelihood of health impairment with a positive outcome.

## 4. Discussion

This study produced quantitative estimates of glycated, oxidized, nitrated, crosslinked, and branched-chain amino acids in healthy subjects and changes in early-stage decline of metabolic, vascular, and renal health. The levels found suggested that for amino acid crosslinks, glucosepane had the highest urinary excretion, and for oxidized amino acids, GSA had the high urinary excretion. The urinary excretion of 3-NT was relatively low. This study also revealed the potential diagnostic utility of urinary BCAAs and FL. Positive likelihood ratios suggested that the use of urinary excretion of BCAA and FL as features in diagnostic algorithms gave small, moderate, and conclusive evidence of increased likelihood of early-stage cardiovascular, metabolic, and renal disease, respectively.

We chose urine analysis as the basis for our health screen. The ease of sample collection and analysis sees urine analysis as a current focus of development in quantitative clinical metabolomics. Compared to plasma or serum, urine has the disadvantage of being less well-buffered than plasma or serum and metabolite levels influenced by the time since previous bladder voiding and food consumption. In the current method, urine ultrafiltrate is mixed with stable isotopic standards in 0.1% trifluoroacetic acid which stabilizes analytes from risk of degradation at neutral and high pH; all are stable at low pH and ambient temperature. A further advantage is that plasma concentrations of glycated, oxidized, and nitrated amino acids are highly dependent on glomerular filtration rate due to related renal clearance whereas urinary concentrations of glycated, oxidized, and nitrated amino acids are not—being related rather to flux of protein glycation, oxidation, and nitration [11, 45]. For these reasons, we chose urine analysis as a preferred sample analysis protocol for health screening related to markers of protein glycation, oxidation, and nitration—although in machine learning analysis, only FL and BCAAs were informative features for subject group classification.

We defined impaired metabolic health herein by reference to detection of impaired glucose tolerance by increased A1C and to insulin resistance represented by the HOMA-IR. This is associated with exposure to increased glucose concentrations as judged by continuous glucose monitoring studies [46]. It was surprising that urinary excretion of glucose-derived FL was not increased in impaired metabolic health, *cf*. the marked increase in urinary excretion in patients with diabetes [47]. This may be due to efficient repair of FL free adduct by fructosamine 3-phosphokinase at levels formed in prediabetes [48] and a relatively large and variable contribution from food which may have increased data dispersion and thereby precluding detection of differences between study groups. Increased glucose exposure was rather reflected in increased urinary excretion of the glucose-dependent glycation-derived crosslink, glucosepane. Glucosepane is formed from the degradation of FL and is not repaired enzymatically, potentially providing greater sensitivity for detection of dysglycemia. In analysis of protein glycation and oxidation adducts in plasma protein in a prospective study, we recently found that levels of glucosepane were the best predictor of development of T2DM [6]. Increased urinary excretion of GSA and CML—formed oxidatively from FL [25]—in early-stage impaired metabolic health may be indicative of oxidative stress. There was also increased urinary excretion of pyrraline which may indicate increased food consumption in this study group. The positive correlations of CML and GSA with pyrraline suggest that urinary increases of these metabolites were linked to increased food consumption. Surprisingly, there was no increase in urinary BCAAs in impaired metabolic health. Increased plasma levels of BCAA were found previously in overweight and obese subjects and correlated positively with insulin resistance [49, 50].

Vascular health impairment was associated with increased urinary excretion of glucosepane. Both glucosepane and CIMT correlated positively with age. Glucosepane is a major protein crosslink and may impact negatively on vascular contractility and function [25].

Renal health impairment was associated with decreased urinary excretion of individual and total BCAAs and also decreased urinary GEEK. The latter is a protein crosslink formed enzymatically by transglutaminases. Increased transglutaminase activity was associated with development of CKD [51]. Our finding is consistent with decreased removal of the GEEK crosslinks produced by increased transglutaminase activity in early-stage decline in renal function. A decrease of urinary excretion of BCAAs in chronic renal insufficiency was found previously—reviewed in [22], linked to both change in tubular transport and decreased interorgan exchange from impaired output by peripheral tissues [52].

In the machine learning-based analysis, we found that combinations of age, BMI with FL, and val, leu, or total BCAA was able to discriminate between healthy controls and subjects with early-stage metabolic, vascular, or renal health decline. Declining metabolic health, referenced to A1C and HOMA-IR, is associated with increased risk of developing type 2 diabetes [53]. Declining vascular health, referenced to increased CIMT, reflects asymptomatic atherosclerosis and increased risk of myocardial infarction and stroke [54]. Declining renal health, referenced to increased serum creatinine and decreased eGFR, is a measure of increased risk of renal disease linked to premature mortality and progression to renal failure [55]. The dominant involvement of subject age is expected as a nonmodifiable risk factor for these health impairments and BMI through association with insulin resistance of impaired metabolic health.

In subjects with impaired metabolic health, BMI correlated positively with urinary excretion of MG-H1, which is maintained when normalised to pyrraline, i.e., corrected for dietary AGE intake. MG-H1 is the major AGE formed from the reactive dicarbonyl metabolite, methylglyoxal. This suggests a link of impaired metabolic health with increased MG exposure, or dicarbonyl stress, for which independent evidence has emerged recently [56]. The failure of urinary excretion of CML and MG-H1 to emerge as diagnostic features for diagnosis of early decline in metabolic and vascular health may be due to a large and variable contribution to urinary excretion of these analytes from digested food, compared to increases of endogenous formation.

Potential utility of measurement of glycated, oxidized, nitrated, and crosslinked amino acids and BCAAs in urine is suggested by the ease of urine sample collection and that damaged amino acids and disturbance in the levels of BCAAs may provide biomarkers of early-stage health decline preceding the development of chronic disease of high prevalence, morbidity, and mortality—diabetes, CVD, and CKD. A urine screening test for early-stage health decline would be a valuable clinical diagnostic asset—particularly when diagnostic assessment for all three early-stage health declines could be made from one analytical run. Screening for prediabetes in overweight and obese adults is considered to be cost effective when linked to subsequent implementation of lifestyle interventions to prevent T2DM [57]. There is profound limited awareness of early-stage CKD with only 9% of people with stage 3 CKD aware of their health impairment [58]—screening for CKD by serum creatinine and deduced estimated GFR (eGFR) is considered not cost effective other than for patients with T2DM and thereby increased risk of CKD [59]. In this initial study, however, we conclude that although there are changes in urinary glycated, oxidized, crosslinked, and branched-chain amino acids in these early-stage health declines, their measurement provides some but limited clinical diagnostic classifications for health screening.

An alternative noninvasive measure of insulin resistance is the ^13^C-glucose test which requires ingestion of a stable isotopically labelled ^13^C-glucose drink and collection of breath samples over the subsequent 6 h for measurement of exhaled [^13^C]/[^12^C]CO_2_ ratio. Our test had similar accuracy and DOR values for detection of insulin resistance without requirement for administration of stable isotope and sample collections over 6 h [60]. An alternative noninvasive method for detection of impaired glucose tolerance is measurement of skin autofluorescence but this is compared poorly, with DOR of 2.7 [61], *cf.* DOR of 25.6 – 27.1 for methods herein. Alternative biomarkers for increased CIMT as an indicator of impaired vascular health are red cell distribution width and serum podocalyxin with DOR values of 3.9 and 3.2, respectively [62, 63]. Our algorithms fared better with values of 5.7 – 6.6. An alternative noninvasive biomarker of impaired renal health is salivary creatinine for which the DOR value for the same classification of CKD as applied herein was 17.8 [64], *cf*. DOR values herein of 34.3 - 78.5. In the diagnostic algorithm for detection of any early-stage health decline studied (metabolic, vascular, and renal health), the negative predictive value of 92% and DOR of 14.7 (Table 6) could provide the basis for screening for the absence of early-stage health decline, after further independent validation.

Potential implementation of this urinary health screening test would be facilitated with further studies to ease access, increase sample analysis throughput, and further validation on one or more independent clinical subject cohorts. Ease of access to the test would be improved through urinary sample donation at primary health care centers. For this, further studies of sample stability during storage and shipment at ambient temperature for urinary analytes of N_*ε*_-fructosyl-lysine (FL) and BCAAs would be required. Studies on sample storage and preanalytical processing validation for unfocussed metabolomic analysis of urine were recently reviewed [65]. For analyte quantitation, sample throughput may be increased with FL, BCAAs, and creatinine analysed in one run by stable isotopic dilution analysis LC-MS/MS [66] or alternatively by ^1^H nuclear magnetic resonance [67]. We estimate minimum sample analysis time would be *ca.* 10 – 20 min and 7 min, for these methods, respectively. For further independent validation of diagnostic outcomes, the assessment of good health versus health impairment (any type) had sensitivity of 81.5%. From statistical considerations based on this and absolute precision of ±5% [68], a validation study requires a minimum of 464 subjects for a 1 : 1 ratio of cases and controls.

We studied early-stage health decline as part of the rationale of the BIOCLAIMS research project and the related concept of “health biomarkers”—see above. There is also a consensus view that if health decline can be detected in the early stages then progression to frank disease may be prevented. This is particularly important for CVD where a first clinical disease event may be fatal and for diabetes and renal failure where there are difficult-to-treat debilitating and life-threatening complications. Also, screening of early-stage health decline could be made more cost effective if assessments of early-stage development of multiple disease are made concurrently.

## 5. Conclusions

- We investigated the urinary excretion of oxidized, glycated, nitrated, crosslinked, and branched-chain amino acids and their association with early-stage decline in metabolic, vascular, and renal health
- We found characteristic changes in early-stage health decline where algorithm features of age, body mass index (BMI), fructosyl-lysine (FL), and branched-chain amino acids (BCAAs) combined to give small, moderate, and conclusive evidence of increased likelihood of early-stage cardiovascular, metabolic, and renal disease, respectively
- After further validation, urinary measurement of FL and BCAAs could help improve ease of access to diagnosis of impaired metabolic health in prediabetes and asymptomatic arterial stenosis and chronic kidney disease where remedial lifestyle and therapeutic intervention may prevent progression to advanced disease and premature mortality

## Figures and Tables

**Table 1:** Glycated, oxidized, and nitrated amino acid metabolites.

Metabolite class	Urinary metabolite	Comment
Glycation	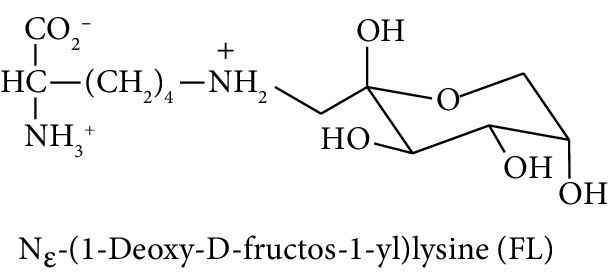	Early-stage glycation adduct [25]. Formed from glucose nonenzymatically and reporting on exposure to increased glucose concentration. Repaired intracellularly by fructosamine 3-phosphokinase [14]. Free adduct absorbed after digestion of food proteins [69].
	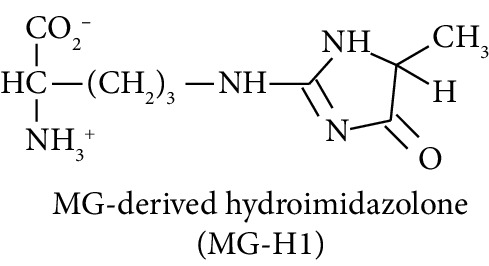	A major quantitative arginine-derived AGE formed from methylglyoxal. Linked to increased fasting and postprandial glucose exposure, insulin resistance, and cardiovascular disease [9, 10, 47, 70]. Free adduct absorbed after digestion of food proteins [9].
	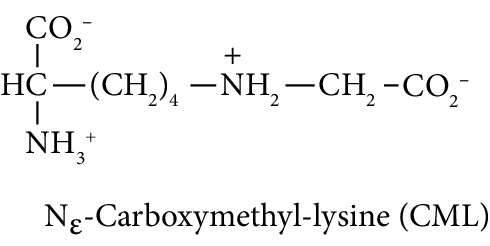	A major quantitative lysine-derived AGE—particularly in food. Formed by the oxidative degradation of FL from and other sources. Free adduct absorbed after digestion of food proteins [71].
	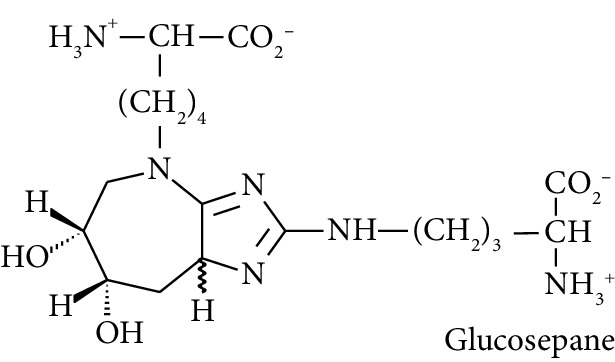	Major quantitative crosslink formed in protein glycation [17].
	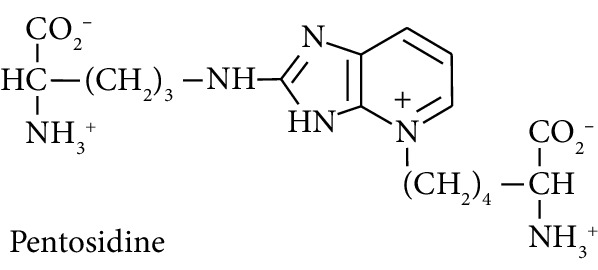	Low level pentose sugar-derived glycation crosslink and intense fluorophore. Considered to reflect pentose phosphate pathway activity [72].
	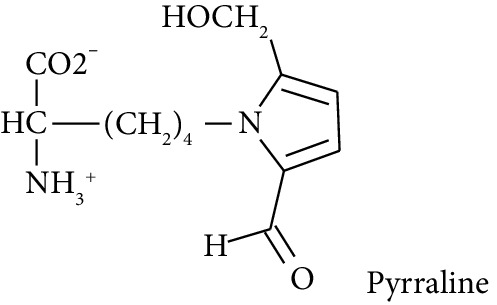	Glucose-derived AGE formed at high temperatures of culinary processing; originating only from food [23, 24].

Oxidation	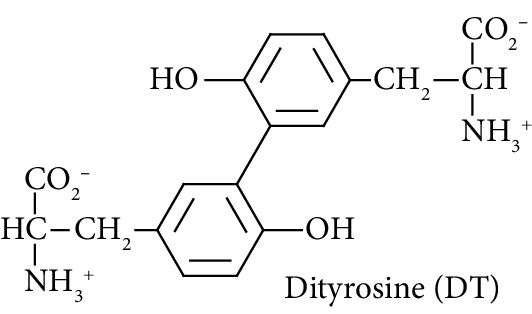	Oxidative crosslink formed spontaneously in oxidative stress and enzymatically by dual oxidase (DUOX) [16, 25].
	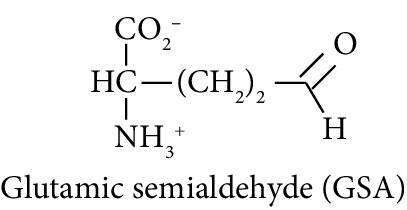	Major “protein carbonyl” formed by the oxidative deguanidylation of arginine and oxidative ring-opening of proline [73].

Nitration	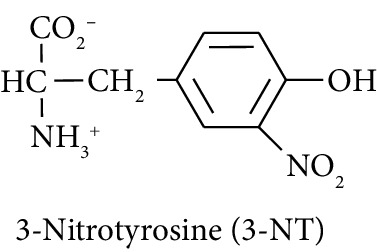	Major proteolysis product of proteins endogenously nitrated by peroxynitrite and nitryl chloride [25, 74].

Transglutaminase-linked crosslink	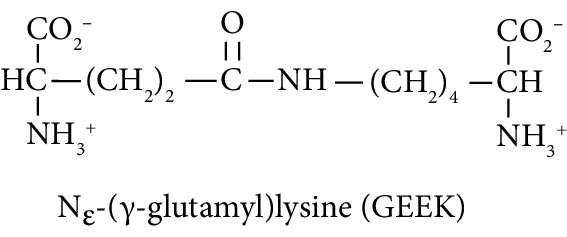	Major protein crosslink formed enzymatically.

Branched-chain amino acids (BCAAs)	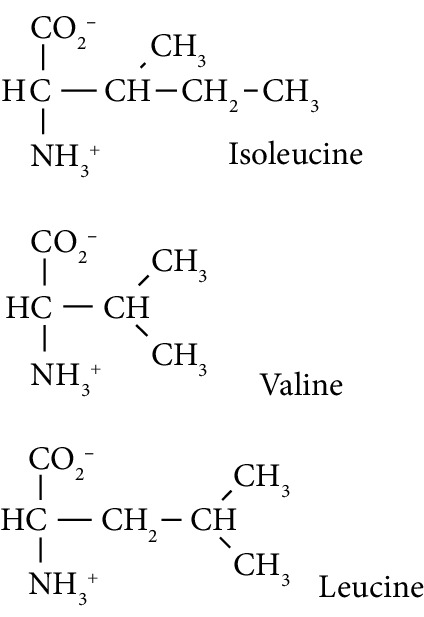	Essential amino acids previously linked to the development of T2DM and CKD [21, 22].

Molecular structures showing ionisation under physiological conditions.

**Table 2:** Mass spectrometric multiple reaction monitoring detection of protein glycation, oxidation, nitration, crosslinks, and branched-chain amino acids.

Analyte group	Analyte	*R*_t_ (min)	Parent ion (Da)	Ion (Da)	CE (eV)	Neutral fragment loss (es)	Internal standard and amount added
Glycation	FL	28.5	291.0	84.3	31	H_2_CO_2_, fructosylamine	[^2^H_4_]FL, 0.3 pmol
	CML	28.5	204.9	130.1	12	NH_2_CH_2_CO_2_H	[^13^C_6_]CML, 0.25 pmol
	MG-H1^†^	11.6 & 12.5	229.2	114.3	14	NH_2_CH(CO_2_H)CH_2_CH=CH_2_	[^15^N_2_]MG-H1, 1.25 pmol
	Glucosepane	16.5	429.2	382.1	38	C_2_H_5_O	[^13^C_6_]Glucosepane, 0.25 pmol
	Pyrraline	17.9	255.2	84.3	23	2-CHO-5-HOCH_2_-pyrrole, H_2_CO_2_	[^13^C_6_,^15^N_2_]Pyrraline, 1.00 pmol

Oxidative damage	Dityrosine	19.9	361.2	315.3	15	H_2_CO_2_	[^2^H_6_]DT, 0.25 pmol
	GSA	32.2	114.0	68.0	15	H_2_CO_2_	[^2^H_3_]AAA, 2.5 pmol

Nitration damage	3-NT	23.2	227.1	181.2	13	H_2_CO_2_	[^2^H_3_]3-NT, 0.25 pmol

TG crosslink	GEEK	9.3	276.1	147.1	12	NH_2_CH(CO_2_H)CH_2_CH=C=O	N*ε*-(*γ*-[^13^C_5_]glutamyl) lysine, 2.5 pmol

BCAA	Leu	27.6	132.3	86.2	10	H_2_CO_2_	[^2^H_3_]Leu, 250 pmol
	Ile	31.5	132.3	86.2	10	H_2_CO_2_	[^13^C_6_]Ile, 250 pmol
	Val	8.2	117.8	72.0	19	H_2_CO_2_	[^2^H_8_]Val, 250 pmol

^†^For MG-H1, *R*_t_ values for the 2 epimers are given. LC-MS/MS was performed as described previously [25, 35] with chromatography using two Hypercarb™ (5 *μ*m particle size, 0.2 × 50 mm and 0.2 × 250 mm) columns, column switching, and elution with 0.1% trifluoroacetic acid (TFA) in water and custom acetonitrile (MeCN) gradient. Different chromatography conditions were used for assay of GEEK: the column was Hypercarb™ (2 *μ*m particle size, 0.2 × 150 mm) with isocratic elution at 0.2 ml/min with 3.75% MeCN and 0.1% TFA in water (solvent A) for 15 min. After each run the column was washed by elution with 50% tetrahydrofuran in 0.1% TFA in water for 20 min and reequilibrated by elution with solvent A at 0.4 ml/min for 15 min. Pentosidine was detected by in-line fluorimetry; excitation wavelength 320 nm, emission wavelength 365 nm [35].

**Table 3:** Clinical characteristics of subjects.

Variable	Healthy controls	Impaired metabolic health	Impaired vascular health	Impaired renal health	Significance (ANOVA/Kruskal-Wallis)
*N*	55	44	58	43	
Age (yrs)	33.3 (26.8 – 41.8)	57.5 (43.9 – 62.1)^∗∗∗^	49.3 (34.1 – 59.0)^∗∗∗^	57.2 (48.3 – 69.7)^∗∗∗^^,††^	<0.001
Gender (M/F)	27/28	22/22	23/35	30/13	
BMI (kg/m^2^)	23.1 ± 2.5	28.9±4.5^∗∗∗^	24.9±3.5^∗∗^^,OOO^	26.3±3.6^∗∗∗^^,OO,†^	<0.001
Smoker (current/ex/never)	13/11/31	8/13/23	9/7/42	2/17/24	
Alcohol intake (mg/day)	4,842 (1,778 – 9,481)	5,048 (1,056 – 10,428)	4,870 (1,851 – 11,658)	5,303 (1,035 – 14,532)	
FPG (mM)	4.79 ± 0.41	5.32±0.58^∗∗∗^	4.92 ± 0.54^OOO^	5.12±0.53^∗∗∗^^,†^	<0.001
A1C (mmol/mol Hb)	34.6 ± 3.1	40.6±2.3^∗∗∗^	37.6±3.0^∗∗∗^^,OOO^	37.7±4.4^∗∗∗^^,OOO^	<0.001
Plasma insulin (IU/ml)	7.00 (4.90 – 9.10)	15.83 (12.49 – 19.00)^∗∗∗^	7.55 (5.60 – 9.48)^OOO^	8.70 (6.20 – 13.65)^∗∗^^,OOO^	<0.001
HOMA-IR (IU/ml/mM)	1.41 (1.11 – 1.96)	3.69 (2.93 – 4.34)^∗∗∗^	1.66 (1.19 – 2.10)^OOO^	1.98 (1.47 – 3.23)^∗^^,OOO,†^	<0.001
Plasma cystatin C (mg/l)	0.755 ± 0.088	0.810±0.104^∗∗^	0.773 ± 0.119	1.346±0.224^∗∗∗^^,OOO^	<0.001
eGFR (ml/min/1.73 m^2^)	88.0 ± 12.5	79.6±11.7^∗∗∗^	84.5 ± 16.2	45.0±8.8^∗∗∗^^,OOO^	<0.001
CIMT (mm)	0.475 ± 0.065	0.572±0.086^∗∗∗^	0.643±0.107^∗∗∗^^,OOO^	0.605±0.070^∗∗∗^	<0.001
Total cholesterol (mmol/l)	4.69 ± 0.66	5.22±1.00^∗∗^	5.38±0.84^∗∗∗^	4.90 ± 0.93^††^	<0.001
LDL cholesterol (mmol/l)	2.61 ± 0.60	3.11±0.88^∗∗^	3.17±0.83^∗∗∗^	2.86 ± 0.95	<0.01
Apolipoprotein-B (g/l)	0.794 ± 0.168	0.988±0.220^∗∗∗^	0.903±0.203^∗∗^^,O^	0.898 ± 0.229^∗^	<0.001
HDL cholesterol (mmol/l)	1.75 ± 0.42	1.42±0.46^∗∗∗^	1.84 ± 0.48^OOO^	1.42±0.49^∗∗∗^^,†††^	<0.001
Apolipoprotein-A1 (g/l)	1.60 ± 0.24	1.54 ± 0.27	1.69 ± 0.30^O^	1.55 ± 0.28^†^	<0.05
Triglycerides (mmol/l)	0.72 ± 0.27	1.51±0.92^∗∗∗^	0.81 ± 0.38^OOO^	1.37±1.09^∗∗∗^^,††^	<0.001
Systolic blood pressure (mmHg)	115 ± 12	126±20^∗∗^	115 ± 12^OO^	123±14^∗∗,††^	<0.001
Diastolic blood pressure (mmHg)	75 ± 7	82±10^∗∗∗^	77 ± 9^OO^	80±9^∗∗^	<0.001
Urinary albumin (mg/24 h)	7.0 (5.1 – 9.2)	7.5 (5.4 – 12.8)	6.7 (4.6 – 9.3)	26.5 (6.6 – 129.4)^∗∗∗^^,OO,†††^	<0.001
Urinary total protein (mg/24 h)	99 (76 – 120)	89 (64 – 111)	89 (72 – 115)	121 (83 – 246)^∗∗^^,OO,††^	<0.01

Data are mean ± SD or median (lower-upper quartile). Significance: ^∗^, ^∗∗^, and ^∗∗∗^, *P* < 0.05, *P* < 0.01, and *P* < 0.001 with respect to heathy controls; o, oo, and ooo, *P* < 0.05, *P* < 0.01, and *P* < 0.001 with respect to impaired metabolic health; and †, ††, and †††, *P* < 0.05, *P* < 0.01, and *P* < 0.001 with respect to impaired vascular health.

**Table 4:** Urinary amino acid biomarkers.

Variable	Healthy controls	Impaired metabolic health	Impaired vascular health	Impaired renal health	Significance (ANOVA/Kruskal-Wallis)
FL (nmol/mg creatinine)	26.5 (17.3 – 39.4)	21.5 (17.1 – 33.9)	23.2 (16.7 – 38.5)	23.9 (19.2 – 29.2)	
CML (nmol/mg creatinine)	15.6 (13.7 – 20.6)	19.6 (15.4 - 25.8)^∗^	16.2 (13.1 – 22.5)^O^	18.3 (14.1 – 21.1)	
MG-H1 (nmol/mg creatinine)	9.75 (5.93 – 15.47)	7.79 (6.53 – 13.68)	8.72 (5.23 – 14.79)	9.68 (7.29 – 11.87)	
Glucosepane (nmol/mg creatinine)	2.84 (2.41 – 3.36)	3.17 (2.64 – 3.95)^∗^	3.19 (2.59 – 4.58)^∗∗^	2.84 (2.44 – 4.21)	<0.05
Pentosidine (nmol/mg creatinine)	0.258 (0.207 – 0.287)	0.266 (0.228 – 0.306)	0.270 (0.212 – 0.315)	0.237 (0.172 – 0.286)	
Pyrraline (nmol/mg creatinine)	9.11 (5.69 – 13.67)	12.5 (7.2 – 16.8)^∗^	11.2 (6.3 – 15.6)	7.2 (5.6 – 10.9)	<0.05
DT (nmol/mg creatinine)	0.053 (0.044 – 0.061)	0.051 (0.043 – 0.061)	0.055 (0.046 – 0.067)	0.052 (0.044 – 0.059)	
GSA (nmol/mg creatinine)	7.45 (5.51 – 8.96)	9.36 (7.03 – 12.16)^∗∗^	7.74 (5.92 – 9.40)^O^	8.08 (6.42 – 9.91)	<0.05
3-NT (nmol/mg creatinine)	0.012 (0.007 – 0.026)	0.011 (0.005 – 0.025)	0.012 (0.006 – 0.022)	0.012 (0.008 – 0.017)	
GEEK (nmol/mg creatinine)	0.422 (0.202 – 0.927)	0.359 (0.222 – 0.619)	0.403 (0.250 – 0.647)	0.277 (0.185 – 0.471)^∗^	
Leu (nmol/mg creatinine)	22.9 (20.6 – 29.0)	24.4 (19.7 – 32.0)	22.8 (19.0 – 27.9)	16.5 (13.9 – 21.0)^∗∗∗^^,OOO,†††^	<0.001
Ile (nmol/mg creatinine)	11.2 (9.2 – 13.1)	11.6 (9.1 – 15.0)	10.8 (8.6 – 13.0)	7.2 (5.8 – 9.0)^∗∗∗^^,OOO,†††^	<0.001
Val (nmol/mg creatinine)	32.7 (29.8 – 37.7)	36.0 (28.6 – 45.1)	29.9 (25.8 – 37.1)^O^	19.6 (15.4 – 26.3)^∗∗∗^^,OOO,†††^	<0.001
BCAA (nmol/mg creatinine)	67.2 (60.3 – 80.8)	73.3 (57.0 – 97.7)	64.6 (56.2 – 77.7)	44.2 (35.9 – 55.0)^∗∗∗^^,OO,†^	<0.001

Data are median (lower-upper quartile) with *N* values as given in Table 2. Significance: ^∗^, ^∗∗^, and ^∗∗∗^, *P* < 0.05, *P* < 0.01, and *P* < 0.001 with respect to heathy controls; o, oo, and ooo, *P* < 0.05, *P* < 0.01, and *P* < 0.001 with respect to impaired metabolic health; and †, ††, and †††, *P* < 0.05, *P* < 0.01, and *P* < 0.001 with respect to impaired vascular health.

**Table:** (a) Algorithm outcome to detect health impairment comparing against good health

Algorithm	Set 1			Set 2		
Features	Age, BMI, FL, and val			Age, BMI, and leu		
Health impairment	Metabolic	Vascular	Renal	Metabolic	Vascular	Renal
Accuracy (%)	83.5 (83.0 – 84.1)	70.5 (69.8 – 71.2)	89.9 (89.4 – 90.4)	84.0 (83.4 – 84.5)	71.6 (70.9 – 72.2)	85.4 (84.9 – 85.9)
Sensitivity (%)	79.1 (78.1 – 89.1)	69.7 (68.6 – 70.7)	87.8 (86.8 – 88.8)	79.9 (78.9 – 81.0)	67.7 (66.5 – 69.0)	80.6 (79.5 – 81.7)
Specificity (%)	87.1 (86.1 – 88.1)	71.4 (70.1 – 72.7)	91.6 (90.9 – 92.4)	87.2 (86.3 – 88.1)	75.6 (74.4 – 76.9)	89.2 (88.3 – 90.1)
Positive likelihood ratio	7.95 (7.24 – 8.65)	2.77 (2.59 – 2.96)	13.2 (12.2 – 14.2)	7.73 (7.09 – 8.36)	3.17 (2.97 – 3.37)	9.56 (8.74 – 10.4)
Negative likelihood ratio	0.24 (0.23 – 0.25)	0.43 (0.41 – 0.44)	0.13 (0.12 – 0.14)	0.23 (0.22 – 0.24)	0.43 (0.41 – 0.44)	0.22 (0.20 – 0.23)
Positive predictive value (%)	83.9 (82.9 – 84.9)	72.6 (71.7 – 73.5)	89.6 (88.8 – 90.4)	83.9 (83.0 – 84.8)	75.2 (74.3 – 76.0)	86.1 (85.2 – 87.1)
Negative predictive value (%)	84.2 (83.6 – 84.8)	69.4 (68.6 – 70.2)	90.9 (90.2 – 91.6)	84.7 (84.1 – 85.4)	69.4 (68.6 – 70.2)	85.6 (85.2 – 86.5)
F-measure	0.81 (0.80 – 0.82)	0.71 (0.70 – 0.72)	0.88 (0.87 – 0.89)	0.82 (0.81 – 0.82)	0.71 (0.70 – 0.72)	0.83 (0.82 – 0.83)
Diagnostic odds ratio	25.6 (23.1 – 28.1)	5.7 (5.3 – 6.1)	78.5 (70.0 – 87.0)	27.1 (24.6 – 29.6)	6.5 (6.1 – 6.9)	34.3 (30.9 – 37.7)

Diagnostic performance data are reported as mean (95% CI) of 100 times repeated 2-fold validation experiments.

**Table:** (b) Algorithm outcome with 2-fold validation to detect health impairment by type using the SVM algorithm comparing against good health

Algorithm	Set 3		
Features	Age, BMI, and BCAA		
Health impairment	Metabolic	Vascular	Renal
Accuracy (%)	84.2 (83.6 – 84.7)	71.8 (71.1 – 72.4)	88.3 (87.8 – 88.9)
Sensitivity (%)	80.1 (79.2 – 81.0)	68.7 (67.6 – 69.8)	85.2 (84.0 – 86.5)
Specificity (%)	87.4 (86.5 – 88.3)	75.0 (73.9 – 76.1)	90.8 (90.0 – 91.6)
Positive likelihood ratio	8.04 (7.35 – 8.73)	3.13 (2.90 – 3.35)	11.6 (10.7 – 12.5)
Negative likelihood ratio	0.23 (0.22 – 0.24)	0.42 (0.40 – 0.43)	0.16 (0.15 – 0.17)
Positive predictive value (%)	84.2 (83.3 – 85.1)	74.8 (74.0 – 75.6)	88.5 (87.6 – 89.3)
Negative predictive value (%)	84.8 (84.3 – 85.4)	69.8 (69.1– 70.5)	89.3 (88.5 – 90.1)
F-measure	0.82 (0.81 – 0.82)	0.71 (0.71 – 0.72)	0.86 (0.86 – 0.87)
Diagnostic odds ratio	27.9 (25.2 – 30.6)	6.6 (6.1 – 7.1)	56.8 (51.1 – 62.5)

Diagnostic performance data are reported as mean (95% CI) of 100 times repeated 2-fold validation experiments.

**Table 6:** Algorithm outcome with 2-fold validation to detect health impairment using the SVM algorithm.

Algorithm	Step 1 – 2-class (good vs. impaired health)	Step 2 – 3 class algorithm (impaired metabolic, vascular, or renal health)		
Features	Age, BMI, FL, and val	Age, BMI, FL, and val		
Health impairment	All	Metabolic	Vascular	Renal
Accuracy (%)	78.2 (77.7 – 78.7)	73.4 (72.9 – 74.0)	68.7 (68.1 – 69.3)	78.1 (75.5 – 78.7)
Sensitivity (%)	81.5 (80.4 – 82.6)	54.9 (53.1 – 56.8)	58.6 (57.2 – 60.0)	67.4 (65.9 – 68.7)
Specificity (%)	77.0 (76.3 – 77.7)	81.5 (80.4 – 82.6)	75.4 (74.2 – 76.6)	82.6 (81.7 – 83.5)
Positive likelihood ratio	3.67 (3.57 – 3.77)	3.43 (3.20 – 3.66)	2.64 (2.51 – 2.77)	4.59 (4.19 – 4.98)
Negative likelihood ratio	0.24 (0.22 – 0.25)	0.55 (0.53 – 0.57)	0.55 (0.53 – 0.56)	0.39 (0.38 – 0.41)
Positive predictive value (%)	57.7 (57.0 – 58.3)	57.8 (56.5 – 59.1)	62.3 (61.3 – 63.2)	63.0 (61.9 – 64.2)
Negative predictive value (%)	91.8 (91.4 – 92.2)	80.9 (80.4 – 81.4)	73.5 (72.9 – 74.0)	85.9 (85.2 – 86.5)
F-measure	0.67 (0.66 – 0.68)	0.55 (0.54 – 0.56)	0.60 (0.59 – 0.61)	0.64 (0.63 – 0.65)
Diagnostic odds ratio	14.7 (14.0 – 15.4)	5.4 (5.0 – 5.8)	4.3 (4.1 – 4.5)	9.8 (8.9 – 10.7)

Diagnostic performance data are reported as mean (95% CI) of 100 times repeated 2-fold validation experiments.

## Data Availability

The data used to support the findings of this study are available from the corresponding author upon request.

## Supplementary Materials

Supplementary Materials The supplementary materials include 4 tables. Supplementary Table 1: correlation analysis of clinical characteristics and urinary analytes: healthy controls. Supplementary Table 2: correlation analysis of clinical characteristics and urinary analytes: impaired metabolic health. Supplementary Table 3: correlation analysis of clinical characteristics and urinary analytes: impaired vascular health. Supplementary Table 4: correlation analysis of clinical characteristics and urinary analytes: impaired renal health.
