# Supplementary material for: Urinary Metabolomic Markers of Protein Glycation, Oxidation, and Nitration in Early-Stage Decline in Metabolic, Vascular, and Renal Health
Source: Oxid Med Cell Longev. 2019 Nov 19;2019:4851323. doi: 10.1155/2019/4851323 (PMC6885816; doi:10.1155/2019/4851323)
Supplement: Supplementary Materials — The supplementary materials include 4 tables. Supplementary Table 1: correlation analysis of clinical characteristics and urinary analytes: healthy controls. Supplementary Table 2: correlation analysis of clinical characteristics and urinary analytes: impaired metabolic health. Supplementary Table 3: correlation analysis of clinical characteristics and urinary analytes: impaired vascular health. Supplementary Table 4: correlation analysis of clinical characteristics and urinary analytes: impaired renal health. [file 4851323.f1.pdf]

Urinary metabolomic markers of protein glycation, oxidation and nitration in early-stage decline in metabolic, vascular and renal health. Jinit Masania *et al.*

SUPPLEMENTARY TABLES

TABLE S1. Correlation analysis of clinical characteristics and urinary analytes: healthy controls.

| | **FL** | **CML** | **MG-H1** | **Pyrraline** | **GSA** | **DT** | **GEEK** | **3-NT** | **Leu** | **Ile** | **Val** |
|---|---|---|---|---|---|---|---|---|---|---|---|
| **FL** | | | | | | | | | | | |
| **CML** | 0.45 | | | | | | | | | | |
| **MG-H1** | 0.84 | 0.43 | | | | | | | | | |
| **Pyrraline** | 0.43 | 0.43 | 0.43 | | | | | | | | |
| **GSA** | | | | | | | | | | | |
| **DT** | 0.44 | | 0.41 | | | | | | | | |
| **GEEK** | | | | | | | | | | | |
| **3-NT** | 0.62 | | 0.72 | | | | | | | | |
| **Leu** | | | | | 0.39 | | 0.47 | | | | |
| **Ile** | | | | | | | 0.47 | | 0.80 | | |
| **Val** | | | | | | | | | 0.82 | 0.74 | |

Spearman correlation coefficients (P<0.05 with Bonferroni correction of 20 applied). The variables compared in correlation analysis were: age, BMI, eGFR, fasting plasma glucose, fasting plasma insulin, A1C, CIMT and urinary analytes: FL, CML. MG-H1, pentosidine, glucosepane, pyrraline, dityrosine, GSA, 3-NT, GEEK, leu, ile and val.

TABLE S2. Correlation analysis of clinical characteristics and urinary analytes: impaired metabolic health

| | | | | | | | | | | | | | |
|---|---|---|---|---|---|---|---|---|---|---|---|---|---|
| **eGFR** | | | | | | | | | | | | | |
| **FL** | | | | | | | | | | | | | |
| **CML** | | 0.46 | | | | | | | | | | | |
| **MG-H1** | | 0.66 | 0.63 | | | | | | | | | | |
| **Pyrraline** | | | 0.66 | 0.52 | | | | | | | | | |
| **GSP** | | | | | | | | | | | | | |
| **GSA** | | | 0.53 | 0.45 | 0.44 | | | | | | | | |
| **DT** | | | | | | 0.45 | | | | | | | |
| **GEEK** | | | | | 0.49 | | | | | | | | |
| **3-NT** | | 0.51 | | 0.71 | | | | | | | | | |
| **Leu** | 0.56 | | | | | | 0.57 | | 0.54 | | | | |
| **Ile** | 0.38 | | 0.56 | 0.57 | 0.48 | | 0.61 | | 0.45 | | 0.79 | | |
| **Val** | 0.50 | | 0.48 | | | | 0.58 | | 0.54 | | 0.89 | 0.81 | |
| | **eGFR** | **FL** | **CML** | **MG-H1** | **Pyrraline** | **GSP** | **GSA** | **DT** | **GEEK** | **3-NT** | **Leu** | **Ile** | **Val** |

Spearman correlation coefficients (P<0.05 with Bonferroni correction of 20 applied).

TABLE S3. Correlation analysis of clinical characteristics and urinary analytes: impaired vascular health.

| | FL | CML | MG-H1 | Pentosidine | GSP | Pyrraline | GSA | DT | 3-NT | Leu | Ile | Val |
|---|---|---|---|---|---|---|---|---|---|---|---|---|
| **FL** | | | | | | | | | | | | |
| **CML** | | | | | | | | | | | | |
| **MG-H1** | 0.74 | 0.68 | | | | | | | | | | |
| **Pentosidine** | | | | | | | | | | | | |
| **GSP** | | | | 0.39 | | | | | | | | |
| **Pyrraline** | | 0.57 | 0.42 | | | | | | | | | |
| **GSA** | | 0.44 | | | | 0.49 | | | | | | |
| **DT** | 0.50 | 0.41 | 0.60 | | | 0.39 | | | | | | |
| **3-NT** | 0.65 | | 0.70 | | | | | 0.44 | | | | |
| **Leu** | | | 0.40 | | | | | 0.49 | | | | |
| **Ile** | | | 0.43 | | | | 0.40 | 0.38 | | 0.72 | | |
| **Val** | | | | 0.42 | | | | 0.42 | | 0.86 | 0.71 | |

Spearman correlation coefficients (P<0.05 with Bonferroni correction of 20 applied).

TABLE S4. Correlation analysis of clinical characteristics and urinary analytes: impaired renal health.

| | | | | | | | | | | | |
|---|---|---|---|---|---|---|---|---|---|---|---|
| **Age** | | | | | | | | | | | |
| **eGFR** | | | | | | | | | | | |
| **FL** | | | | | | | | | | | |
| **CML** | | | | | | | | | | | |
| **MG-H1** | | | 0.46 | 0.54 | | | | | | | |
| **GSP** | 0.46 | | | | | | | | | | |
| **Pyrraline** | | | | 0.50 | 0.55 | | | | | | |
| **3-NT** | | | | | 0.71 | | | | | | |
| **Leu** | | 0.60 | | | | | | | | | |
| **Ile** | | 0.56 | | | | | | | 0.83 | | |
| **Val** | | 0.42 | | | | | | | 0.79 | 0.86 | |
| | **Age** | **eGFR** | **FL** | **CML** | **MG-H1** | **GSP** | **Pyrraline** | **3-NT** | **Leu** | **Ile** | **Val** |

Spearman correlation coefficients (P<0.05 with Bonferroni correction of 20 applied).
